# Bactericidal and Bioactive Dental Composites

**DOI:** 10.3389/fphys.2018.00103

**Published:** 2018-02-16

**Authors:** Xanthippi Chatzistavrou, Anna Lefkelidou, Lambrini Papadopoulou, Eleni Pavlidou, Konstantinos M. Paraskevopoulos, J. Christopher Fenno, Susan Flannagan, Carlos González-Cabezas, Nikos Kotsanos, Petros Papagerakis

**Affiliations:** ^1^Department of Orthodontics and Pediatric Dentistry, University of Michigan, Ann Arbor, MI, United States; ^2^Department of Pediatric Dentistry, Aristotle University of Thessaloniki, Thessaloniki, Greece; ^3^Department of Geology, Aristotle University of Thessaloniki, Thessaloniki, Greece; ^4^Physics Department, Aristotle University of Thessaloniki, Thessaloniki, Greece; ^5^Biologic and Materials Sciences, School of Dentistry, University of Michigan, Ann Arbor, MI, United States; ^6^Cariology, Restorative Sciences and Endodontics, University of Michigan, Ann Arbor, MI, United States

**Keywords:** antibacterial activity, bioactivity, biofilm, *Streptococcus mutans*, silver, bioactive glass, resin composite, total bond strength

## Abstract

**Aim:** Antimicrobial and bioactive restorative materials are needed to develop a bacteria free environment and tight bond with the surrounding tissue, preventing the spread of secondary caries and thus extending the lifetime of dental restorations. The characteristic properties of new dental bioactive and antibacterial composites are presented in this work. The new composites have been microstructurally characterized and both long and short term properties have been studied.

**Methods:** The Ag-doped sol-gel derived bioactive glass (Ag-BG) was incorporated into resin composite in concentrations 5, 10, and 15 wt.%, to fabricate new Ag-doped bioactive and antibacterial dental composites (Ag-BGCOMP). The microstructural properties and elemental analysis of the developed Ag-BGCOMP was observed. The total bond strength (TBS) was measured immediately and after long term of immersion in medium using microtensile testing. The capability of Ag-BGCOMPs to form apatite layer on their surface after immersion in Simulated Body Fluid (SBF) as well as the bacteria growth inhibition in a biofilm formed by *Streptococcus mutans* (*S. mutans*) were evaluated.

**Results:** Homogeneous distribution of Ag-BG particles into the resin composite was observed microstructurally for all Ag-BGCOMPs. The TBS measurements showed non-statistically significant difference between control samples (Ag-BG 0 wt.%) and Ag-BGCOMP specimens. Moreover, the total bond strength between the surrounding tooth tissue and the material of restoration does not present any statistically significant change for all the cases even after 3 months of immersion in the medium. The bioactivity of the Ag-BGCOMPs was also shown by the formation of a calcium-phosphate layer on the surface of the specimens after immersion in SBF. Antibacterial activity was observed for all Ag-BGCOMPs, statistically significant differences were observed between control samples and Ag-BGCOMPs. Accordingly, the number of dead bacteria in the biofilm found to increase significantly with the increase of Ag-BG concentration in the Ag-BGCOMPs.

**Conclusions:** New resin composites with antibacterial and remineralizing properties have been manufactured. Characterization of these materials provides a rationale for future clinical trials to evaluate clinical benefits and outcomes in comparison with currently used dental materials.

**Significance:** The new developed composites could ultimately prevent restoration failure and could advance patients' wellbeing.

## Introduction

The number of dental caries occurring in treated and restored teeth is very high, in some cases up to 50–60% (Imazato, 2003; Fan et al., 2011; Melo et al., 2013). The failure of composite resin restorations is mostly attributed to secondary caries (Sarrett, 2005; Pereira-Cenci et al., 2009, 2013; Mohamed Hamouda, 2012; Wiegand et al., 2014). In the United States, half of all restorations are replacements of failed restorations, with resin composites showing the higher failure rates. The micro-gap at the interface between the restoration and the prepared cavity leads most commonly to the occurrence of secondary decay (Xie et al., 2011). Tooth structure is demineralized following bacteria invasion, such as *S. mutans*, when fermentable carbohydrates are present (Xie et al., 2011). Moreover, considering the tissue-saving approach, where a more conservative strategy of caries removal is nowadays suggested, it is expected that more affected tissue will remain and possibly will harbor more residual bacteria (Imazato et al., 2006; Esteves et al., 2010). This highlights the need for the development of more effective antibacterial and bioactive restorative materials that can prevent colonization of bacteria and secondary decay (Yoshida et al., 1999a,b). New materials capable of diminishing cariogenic bacteria such as *S. mutans*, which has been observed to adhere on restorative materials, are needed (Willershausen et al., 1999). This approach could potentially prevent recurrent decay and even allow tissue-saving removal of caries and influence the micro leakage, which is directly correlated to pulpal inflammation beneath cavities *in vivo* (Tobias, 1988; Beyth et al., 2007; Imazato et al., 2012). In the last case the use of antibacterial materials could also provide an additional treatment by suppressing residual infection and increase the survival of the restored tooth especially in minimally invasive approaches (Imazato et al., 2006).

The currently available commercial composites have little if any antibacterial action (Matalon et al., 2004, 2009; Rathke et al., 2010). This fact is inevitable as none of the components of the resin composites show bactericidal effects against oral bacteria at the concentrations present in the materials (Beyth et al., 2007). On the contrary, it has been suggested that composites enhance cariogenic bacterial growth via the release of ethylene glycol dimethylacrylate and triethylene glycol dimethacrylate (Schmalz et al., 2004; Busscher et al., 2010; Wang et al., 2014). In the case of composites that have been designed to release fluoride (compomers), mostly because of the remineralizing effects (Hotwani et al., 2013), the released amount of fluoride has been measured as too low and not enough to inhibit a significant bacterial growth (Brambilla et al., 2005). It has been suggested that fluoride antibacterial effect cannot last long and mostly lost after 24 h (Matalon et al., 2003, 2006, 2009).

A number of methods are reported in the literature where antibacterial agents have been incorporated in to restorative materials to induce antibacterial action (Chen et al., 2012). There has been special interest in releasing or slow-releasing antibacterial agents with low molecular weight, including ions such zinc, silver or antibiotics, iodine, and chlorhexidine (Xie et al., 2011; Imazato et al., 2012; Weng et al., 2012a,b; Wiegand et al., 2014; Chatzistavrou et al., 2015). Immobilized antibacterial components are also used to add antibacterial properties in the material (Xie et al., 2011; Imazato et al., 2012). The method of immobilization has the advantage of long lasting antibacterial properties (Imazato et al., 2012), however they lack of strong and remote antibacterial action. Very often antibacterial materials with immobilized agents consists of polymers containing quaternary ammonium or phosphonium salts or a combined incorporation of quaternary ammonium and polyethylenimine nanoparticles (Rolland et al., 2011). Silver compounds are also well known to possess antibacterial properties without developing bacterial resistant strains (Peng et al., 2012). New dental composites incorporating quaternary ammonium dimethacrylate (QADM) and silver nanoparticles (AgNP) have been manufactured and observed to inhibit *Streptococcus mutans* (*S. mutans*) (Zhang et al., 2013; Cheng et al., 2018). Combined antibacterial and regenerative action have been the ultimate aim of the new generation of dental composites, and considerable systematic work has been done toward this. Calcium phosphate nanoparticles have been incorporated into composites in addition to QADM and AgNP to induce regenerative properties (Cheng et al., 2012a,c). Calcium-fluoride and chlorhexidine or a new sol-gel derived Ag-doped bioactive glass, are also some of the new components in novel dental materials development aiming to enhance remineralization, regeneration and bactericidal properties (Cheng et al., 2012b; Chatzistavrou et al., 2015).

Although a significant amount of research has been done on developing new antibacterial and bioactive restorative materials for preventing recurrent decay (Chen et al., 2012), most of these new materials show compromised physical properties (Takahashi et al., 2006; Yesilyurt et al., 2009). In particular, the changes in the composition or the leaching of particles that could induce antibacterial action may affect material's mechanical properties and turn it to an unsuitable restorative material or restrict its use to non-loadbearing areas (Takahashi et al., 2006; Yesilyurt et al., 2009). Additional issues may be the changes in material's adhesion property to the surrounding tooth as well as the changes in color, which may allow the use of the material only to posterior restorations (Fan et al., 2011).

In this work new antibacterial and bioactive dental composites have been manufactured and characterized regarding the short and long term properties. The incorporation of a Ag-doped bioactive glass in the resin composite induces remineralizing and antibacterial properties, while the total bond strength of the new Ag-BGCOMP composites is not significantly affected, either immediately after treatment or after long term of immersion in medium.

## Materials and methods

### Fabrication of Ag-BGCOMPs

Specimens with 0, 5, 10, and 15 wt.% of Ag-BG within the flowable composite (Ivoclar Vivadent, Tetric EvoFlow® Filling Material A1, United States and Canada) were manufactured as previously described in detail (Sauro et al., 2013; Chatzistavrou et al., 2014). The Ag-BG in powder form with particle size of < 20μm was incorporated manually. Samples without Ag-BG incorporation, consisted only by composite resin were used as controls (controls: Ag-BG 0 wt.%). Cured disc samples (100 mgr) were prepared using Teflon molds (10 mm diameter and 2 mm thick) following the manufacturer's instructions (two curing cycles of 10 s with halogen curing light [Valo, Ultradent (South Jordan, UT)] operating with a wavelength of 400–500 nm and an intensity of about 1,000 mW/cm^2^. Both top and bottom surfaces were exposed to light. A clear plastic strip was placed over the Teflon molds to prevent the formation of resin rich layer after curing and a glass slide 1 mm thick was placed over the plastic strip to allow standardization of the sample thickness and the distance from sample surface to the curing light-tip.

### Microstructural properties

The microstructural properties and elemental analysis of the developed Ag-BGCOMP was observed using Scanning Electron Microscopy with X-ray microanalysis (SEM-EDS) (JEOL J.S.M. 840A, Tokyo, Japan) on cross sections of specimens embedded in epoxy resin.

### Mechanical properties

The Microtensile test method was used to measure the total bond strength (μTBS) of dentin, as it is considered an appropriate method to characterize the mechanical properties of the Ag-BGCOMPs (Chatzistavrou et al., 2012). In particular, third molars were potted along the long access of the tooth and cut mid-coronally to expose the dentin. The teeth were sanded with 320 grit paper, etched for 15 s using 35% phosphoric acid etchant, blotted dry until they were slightly moist, and then bonded with commercial bonding agent (AdheSE®, Ivoclar Vivadent) as well as ultimately, layered with the manufactured composite formulations. Specimens of 1 × 1 mm in transverse cross-section were cut using a hard tissue microtome along the long axis of the tooth, obtaining matchstick-shaped beams and creating microtensile samples.

The specimens were immediately tested for the μTBS test before immersion as well as after 1 month and 3 months of immersion in culture medium in order to observe the short and long term mechanical properties under conditions that can affect the bonding with the surrounding tissues. This testing procedure was performed using customized microtensile fixtures on a testing set-up comprising a LAC-1 (high speed controller single axis with built-in amplifier) and LAL300 linear actuator that had a stroke length of 50 mm.

### Apatite phase formation

The formation of the apatite phase on the surface of the new composites was observed after immersion of specimens (*n* = 4, from each group) in SBF and incubation at 37°C under shaking conditions. The immersion medium was renewed every other day. The surface of the samples was observed after 7 and 14 days of immersion using the AMRAY 1910 Field Emission Scanning Electron Microscope (FEG-SEM).

### Biofilm formation

**Figure 1:**
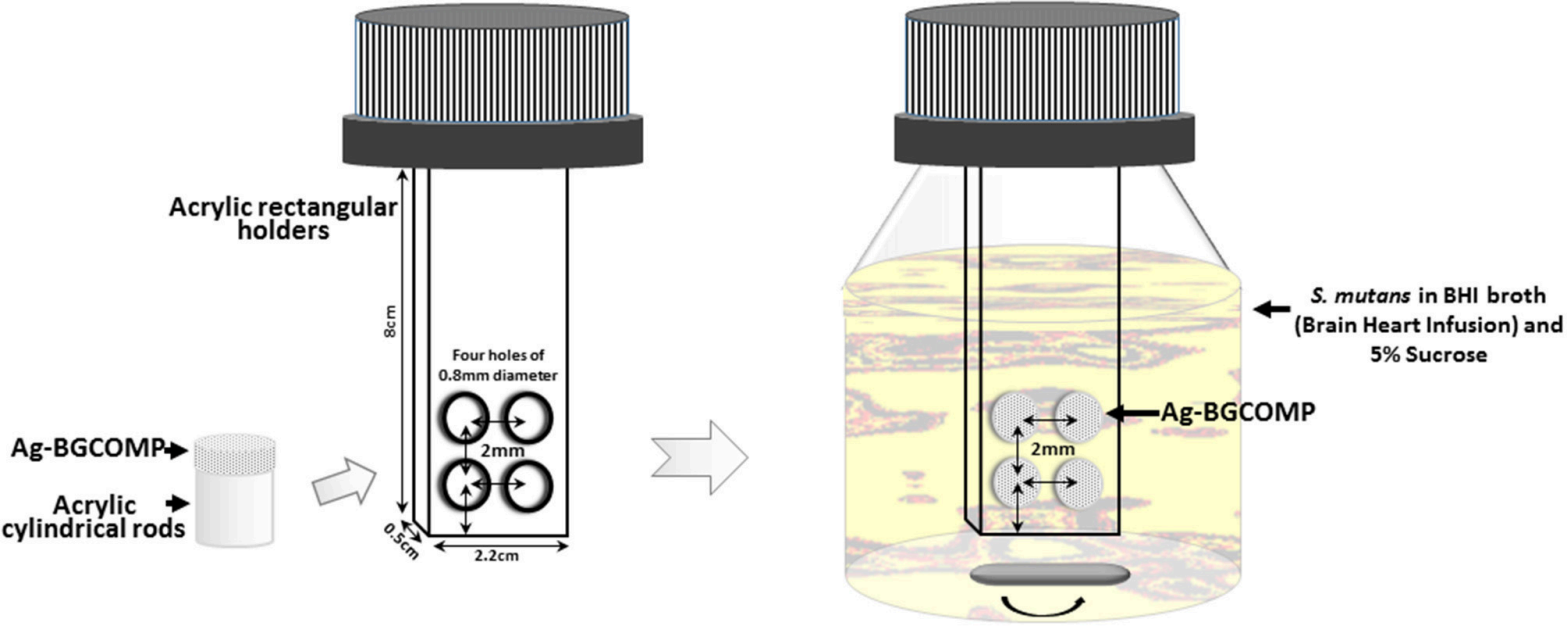
Schematic illustration of the set up system for biofilm formation by *S.mutans* on the surface of Ag-BGCOMP specimens after 48 h of culture.

Biofilms were formed on the surface of the specimens using single bacterial strain (*S.mutans* UA159) following a specific protocol. An acrylic rectangular shaped holder (8 × 2.2 × 0.5 cm) with four cylindrical open areas at the bottom of it with 0.8 mm diameter each, placed in specific distance forming a square (Figure 1). Cylindrical specimens of Ag-BGCOMP were mounted on the top of 0.8 mm diameter acrylic rods, which fit in the open cylindrical areas. Each holder carried four samples of each group. The holders were attached with epoxy resin in plastic bottle caps. The whole construction was subjected to ethylene oxide sterilization. Subsequently, 12 bottles with 80 mL BHI broth (Brain Heart Infusion, Hach, Loveland, CO, USA) supplemented with 5% sucrose were autoclaved (Figure 1).

Gram stain and catalase tests were performed on *S. mutans* UA159 grown in BHI medium. The culture was adjusted to an optical density at 600 nm (OD_600_) of 0.6, which is equivalent to 1 × 10^6^ cells/ml. An aliquot of 5 μl was diluted in 5 ml BHI. Then, 80 μl of the previous dilution were inserted in each of the four bottles with 80 ml BHI supplemented with 5% sucrose. The acrylic sample holders with the Ag-BGCOMP specimens attached were inserted into the bottles and kept at 37°C up to 48 h under stirring conditions (at 150 rpm) for biofilm formation. The medium was replaced at 8 and 16 h. After incubation samples washed four times with 10xPBS for 3 min each time.

The biofilms formed on the surface of the specimens (*n* = 12 for each group) were observed by SEM (AMRAY 1910) and Confocal Laser Scanning Microscopy (CLSM). Samples observed by CLSM were stained by Live/Dead staining (Baclight Viability Kit L7012, (Molecular Probes, Inc.) and the observation was done using a Leica Inverted SP5X Confocal Microscope System with 2-Photon FLIM at 500 and 550 nm, x65 magnification. The images were analyzed by Image J software.

### Long term antibacterial activity

The evaluation of the long term bactericidal properties was performed by collecting the extracts of the specimens after being immersed in PBS for 22 days. A single colony of two different bacteria strains (*Lactobacillus casei* ATCC 15008 and *S. mutans* ATCC 25175) was inoculated separately in nutrient broth and grown overnight at 37°C. After adjusting to an optical density equivalent to 10^8^ cells per ml in PBS, sequential tenfold dilutions were added to tubes containing equal volumes of the extracts. The effect of the materials' extracts on bacterial growth was assayed by colony forming units (CFU) on nutrient agar plates after 24 h of growth.

### Statistical analysis

The sample size for each case of Ag-BGCOMP composite for the mechanical test was calculated based on prior data indicating that the difference in the μTBS of dentin is normally distributed with a SD no larger than 4.19 (Lenzi et al., 2013). Assuming a hypothesized effect size of no < 4.45 MPa (Lenzi et al., 2013), at a significance level of 5%,with a statistical power of 90%, a power analysis based on paired *t*-test indicates a sample size of 11 would be sufficient (power and sample size calculations, version 3.0.43, 2009). The statistical analysis of the results from the mechanical tests (Figure **4**) was performed using the Student's *t*-tests (two-tailed) and ANOVA (one-way) using SigmaStat Software (level of significance *p* < 0.05).

## Results

### Microstructural properties

**Figure 2:**
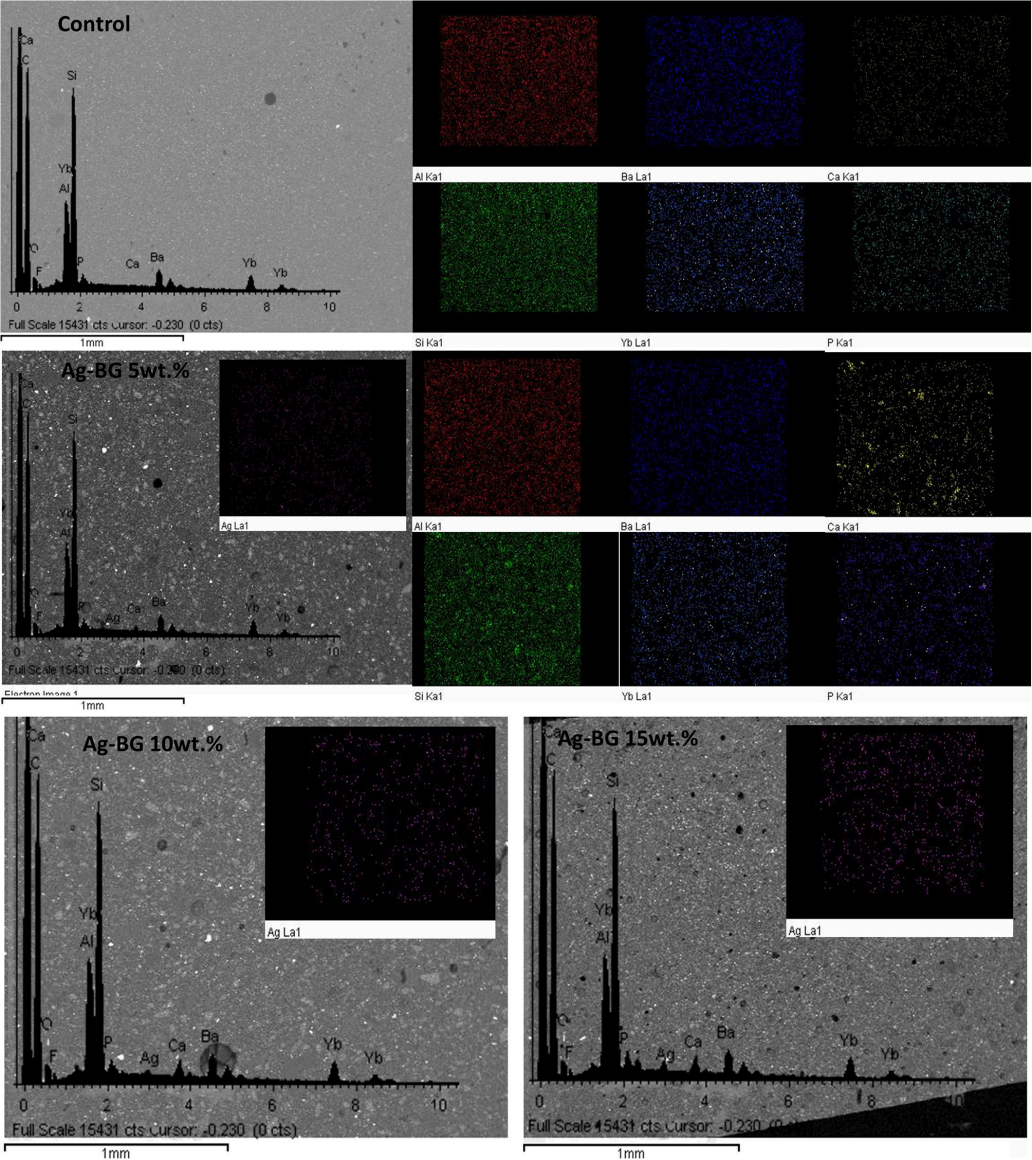
SEM images and elemental mapping analysis of the cross sections of control and Ag-BGCOMP samples.

The elemental mapping analysis on the cross section of the samples reveals a homogeneous distribution of all elements in the resin matrix. The amount of the observed silver ions increases with the increase of Ag-BG in the composites (Figure 2).

**Figure 3:**
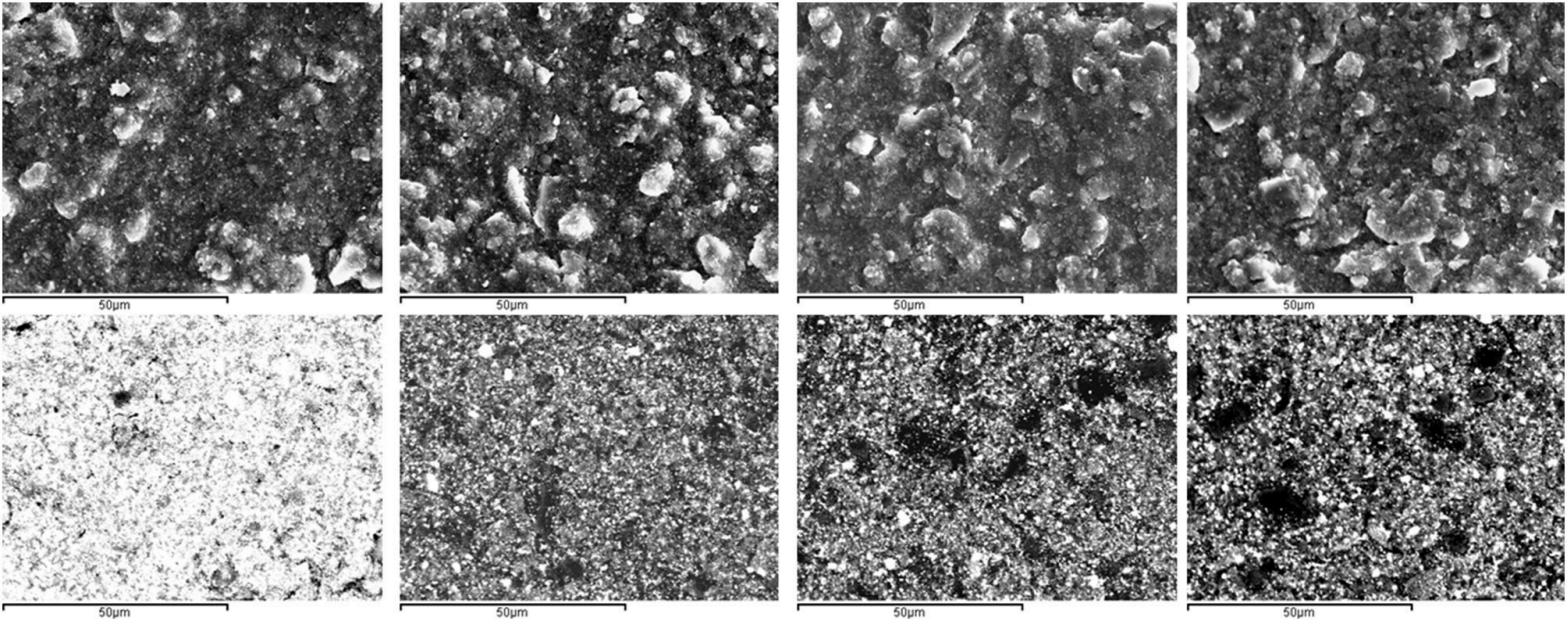
SEM and backscattered images from the surface of control and Ag-BGCOMP samples, showing the surface morphology and qualitatively the Ag-BG distribution.

Surface images and backscattered (Figure 3) show the surface morphology. The distribution of the darkest areas in the backscattered images reveals the incorporation of Ag-BG particles in Ag-BGCOMP composites.

### Mechanical properties

**Figure 4:**
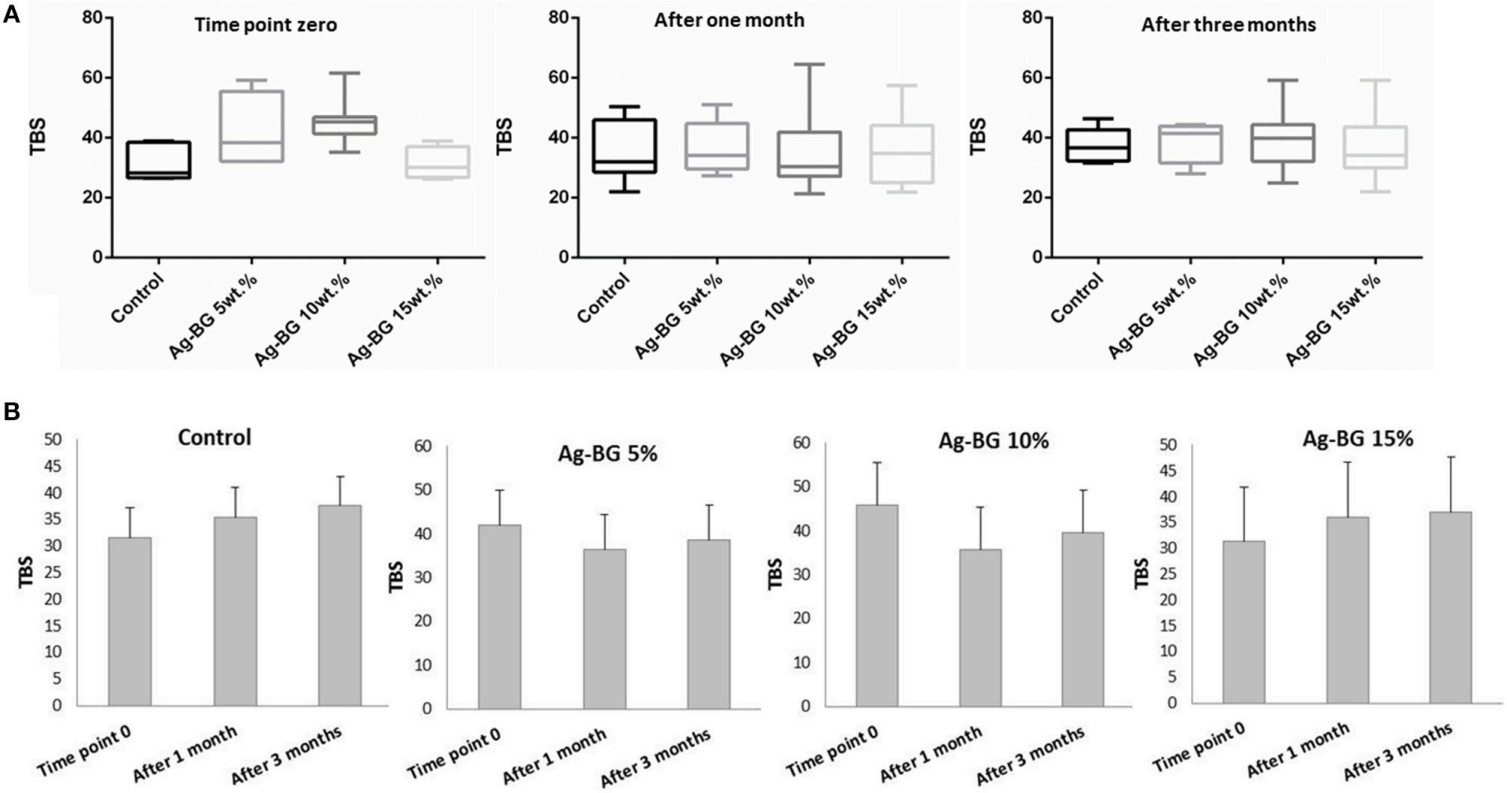
TBS values of control and Ag-BGCOMP samples (Ag-BG in 5, 10, and 15 wt%) measured immediately after teeth treatment and after some time of immersion in medium (1 and 3 months). **(A)** Comparison between groups at different time points and **(B)** comparison between different time points for each group.

The microtensile tests show a non-significant difference on the TBS values between control samples and all the Ag-BGCOM samples (5, 10, and 15 wt%). This is the case for tests performed on treated teeth immediate after the treatment or after immersion in medium for a period of time (Figure 4A).

The bond strength value of each resin composite in all the cases does not significantly change between the immediate test and the those after immersion in medium for long period of time (Figure 4B).

### Remineralization

**Figure 5:**
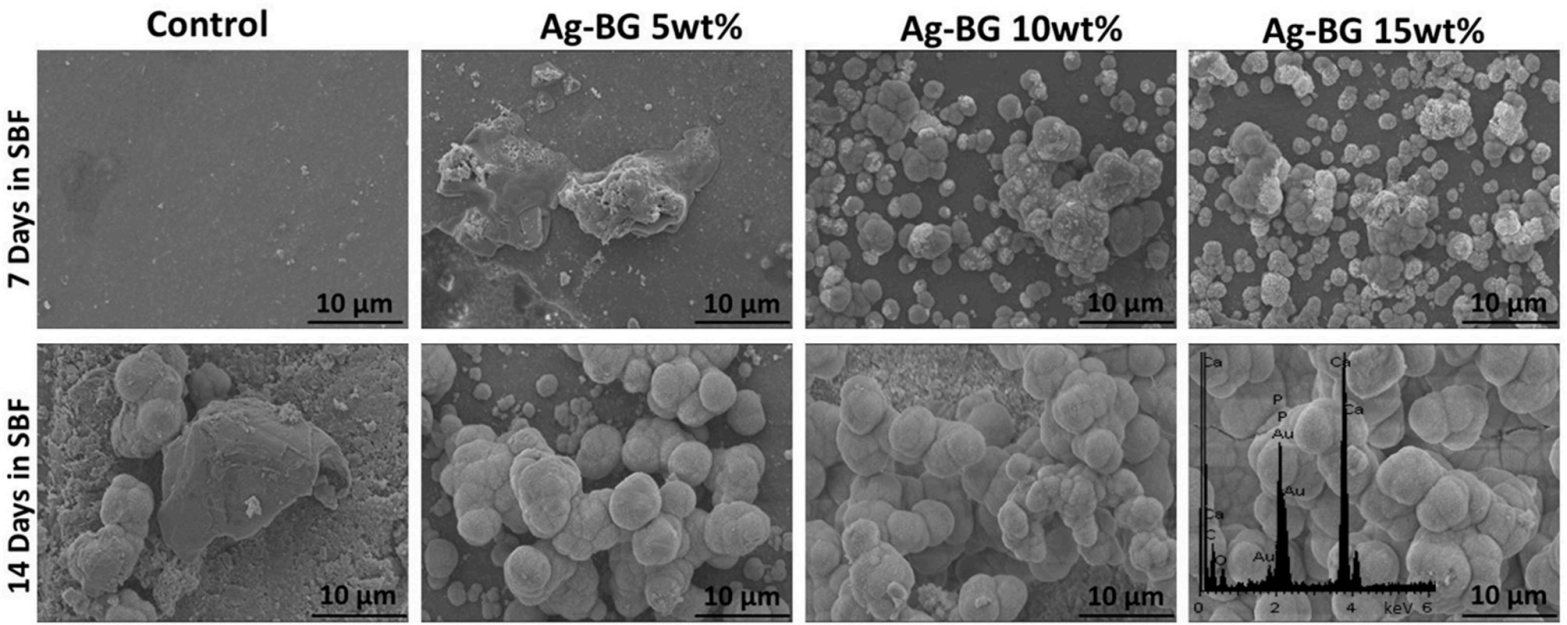
SEM images of the surface morphology of the Ag-BGCOMP specimens after 7 and 14 days of immersion in SBF.

Formation of an apatite-like phase is observed on the surface of the Ag-BGCOMP specimens with 10 and 15 wt% after 7 days of immersion in SBF (Figure 5). SEM images present the morphology the deposited apatite-like phase, while the EDS analysis confirms that Ca-P are the main elements observed in the formed new phase with Ca/P ratio ~1.65. All Ag-BGCOMP specimens present the deposition of an apatite-like phase on their surface after 14 days of immersion in SBF. The amount of the deposited phase seems to get increased by the increase of Ag-BG particles in the composite.

### Inhibition of bacterial growth and biofilm formation

**Figure 6:**
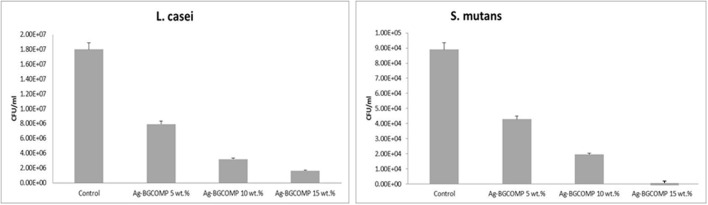
Colony Forming Units per ml for *L. casei* and *S. mutans* after culture with the 22 days extracts of the specimens.

Extracts were collected from the specimens after 22 days of immersion in PBS. The long term bactericidal properties of the extracts against two different bacteria strains (*L. casei* and *S. mutans*) were observed. Measuring the colony forming unit per ml for each case, it is observed a significant bacteria inhibition after culture with the extracts of the 22nd day, for all Ag-BGCOMP specimens compared to the control specimens (Figure 6). This antibacterial action gets even stronger when the Ag-BG amount in the composites is higher than 10 wt.%.

**Figure 7:**
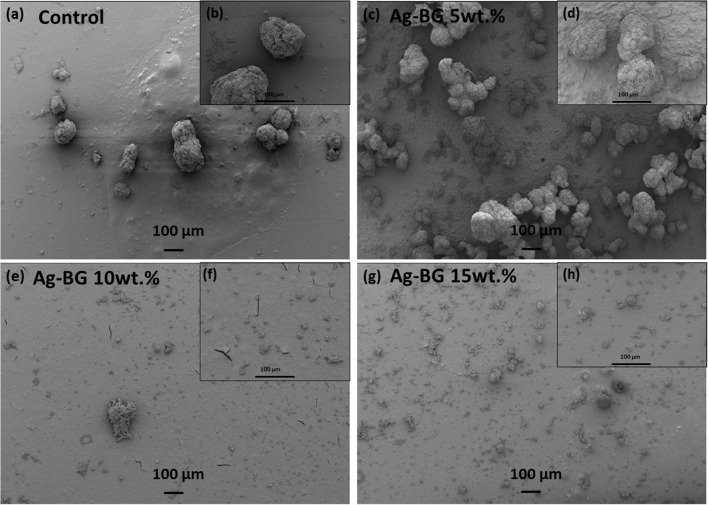
SEM images of the biofilm formed on the surface of the specimens. Images **(b,d,f,h)** show in higher magnification, the biofilm morphology in detail. **(a,c,e,g)** are lower magnification images.

The morphology of the biofilm formed on the surface of the resin specimens after 48 h in culture with *S. mutans* is presented in the Figures 7 a,c,e,g. Images of higher magnification Figures 7b,d,f,h present the size of the formed bacteria aggregations. The size of bacteria aggregations is significantly high in the case of biofilm formed on the surface of control samples and Ag-BGCOMP specimens with 5 wt%. The aggregates have spherical morphology with diameter around 100 μm or smaller. The increase of the Ag-BG concentration in the specimens decreases the size of aggregations. Spherical aggregates with diameter around 20 μm or smaller are formed on the surface of Ag-BGCOMP with 10 and 15 wt.%.

**Figure 8:**
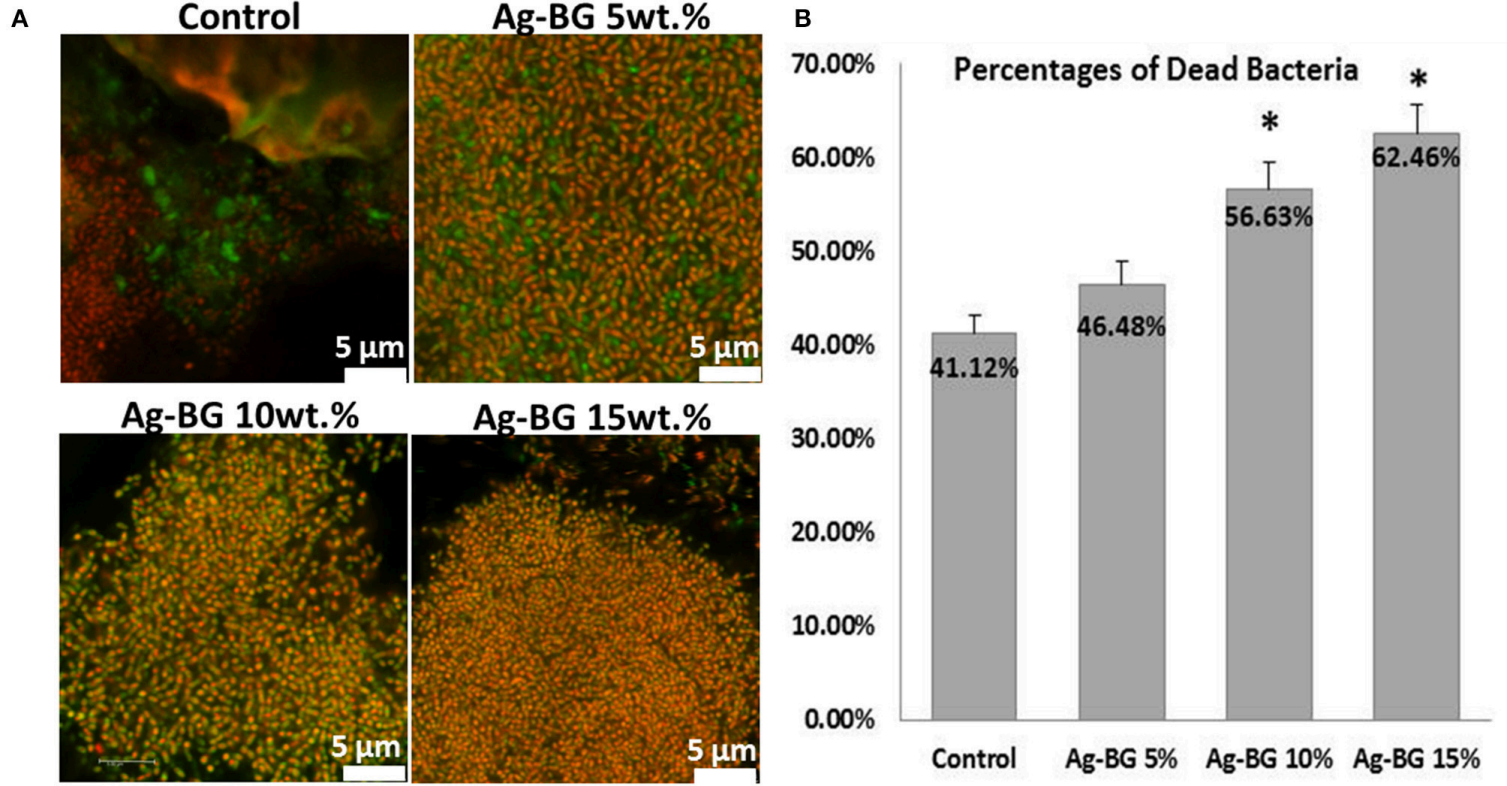
CLSM representative images of the live and dead bacteria in the biofilms formed on the surface of the Ag-BGCOMP specimens with different concentrations in Ag-BG **(A)**. Results from the quantitative analysis of the images showing the percentage of the dead bacteria in the biofilm for each group **(B)**. ^*^shows the statistical significant difference between the groups.

Confocal images present the amount of alive (green) and dead (red) bacteria in the biofilm formed on the surface of the specimens after 48 h of culture (Figure 8A). The quantitative analysis (Figure 8B) of the images confirm that the number of dead bacteria is significantly higher when the amount of Ag-BG is 10 wt.% and higher. For lower concentrations of Ag-BG in the Ag-BGCOMP specimens there is no significant difference in the number of the dead bacteria compared to the control samples.

## Discussion

This study evaluated the properties of Ag-BGCOMP in terms of homogeneity, bioactivity and anti-bacterial effects against S. mutans.

Regarding the homogeneity of the Ag-BGCOMP, microstructural observation indicated that the amount and the particle size of the incorporated Ag-BG were in the right range so that a homogeneous distribution can be achieved manually within the resin composite. The representative backscattered images further support this observation. Therefore, we suggest that addition of Ag-BG in to dental composites is a feasible procedure in order to obtain dental composites with anti-bacterial properties and biological activity.

Regarding the biological activity of the Ag-BGCOMP, we found enhanced remineralizing properties compared to controls through increased formation of apatite phase. In fact, the remineralizing capability of the Ag-BGCOMP is confirmed with the formation of a calcium-phosphate apatite-like phase on the surface of the specimens after more than 14 days of immersion in SBF. This observation is also the case for the specimens with low concentrations in Ag-BG (5 wt.%), while for those with higher concentrations in Ag-BG the amount of the formed phase increases and the developed layer observed denser and possible thicker. The polymerization conversion of Ag-BGCOMP specimens after curing process does not eliminate the bioactive behavior of Ag-BG regarding the capability to form as apatite phase. On the contrary, the incorporated Ag-BG induces bioactivity to the resin composites Ag-BGCOMP. Furthermore, during the apatite formation it is expected that the precipitated SiO_2_-rich layer with the deposited Ca^2+^ and ${\text{PO}}_{4}^{3--}$ species may further favor the formation of a high molecular weight complex (Ca/P–MMPs). This complex structure can ultimately obstruct the enzymatic degradation of metalloproteinases within the hybrid layer (Kremer et al., 1998). Thus, the hydrolysis of both collagen and resin components could be minimized or eliminated due to the remineralization process on the surface of Ag-BGCOMP. Moreover, the released calcium and phosphate ions from the Ag-BGCOMP substrates could show therapeutic remineralizing effects at the tooth lesion areas.

Regarding, the mechanical properties of the Ag-BGCOMP, we found no significant differences after Ag-BG addition compared to control samples even after long period of time being immersed in medium. The total bond strength (TBS) value of the Ag-BGCOMP samples does not present any significant difference compare to the respective value of the control, although based on our previous work a slightly lower degree of polymerization conversion of the resin is expected due to the incorporation of the Ag-BG particles (Kattan et al., 2015). The “time point zero” diagram (a) of Figure 4 indicates a tendency where Ag-BGCOMP samples with low concentrations of Ag-BG (5 and 10 wt.%) exhibit a slightly higher bonding strength than the control samples, while for higher concentrations of Ag-BG this is not the case. This tendency could be attributed to the incorporated Ag-BG particles that act as reinforcement material at low concentrations and as a “filler-like” component at higher concentrations. However this is statistically insignificant. This is also the case for both treated teeth and tested either immediately after treatment or after immersion in medium for long period of time (tested after 1 and 3 months). These results indicate that the adhesion property of the resin composite to the surrounding tooth does not change even after Ag-BG incorporation. The incorporated antibacterial and bioactive particles have been in size and amounts that cannot significantly disrupt the resin composite network and can retain the mechanical characteristics of the control samples. Treated teeth were immersed in medium for different periods of time in order to evaluate the results of the ion leaching process on the mechanical properties. No compromised mechanical properties were observed due to the expected leaching process. It is anticipated that this behavior occurs due to the simultaneous remineralizing process which follows the ion leaching. The remineralization could enhance the bonding with the surrounding dental tissues, retaining the physical properties of the material. This is more obvious at Ag-BGCOMP samples with Ag-BG 15 wt. %, where there is a slight increase of reinforcement after immersion. On the other hand, Ag-BGCOMP samples with lower concentrations of Ag-BG reveal a “loss” of reinforcement after immersion. However, it is not a statistically significant tendency.

Finally, the inhibition of biofilm formation combined with long lasting antibacterial action could potentially suggest the suitability of Ag-BGCOMP for dental applications. More specifically, there is significant difference on the size of the bacteria aggregation between the control, Ag-BGCOMP 5 wt% specimens and those with higher concentrations in Ag-BG (Figure 6). This observation may indicate a lower number of bacteria being alive, attached and organized in smaller aggregates on the surface of those samples with Ag-BG 10 and 15 wt.%. Bacteria are able to attach and form a biofilm on the surface of all specimens. However silver ions are expected to be released from the substrate as immersion time increases. The concentration of the released ions should increase with the increase of Ag-BG in the composites, leading to a significant increase of the number of dead bacteria in the biofilm. An expected result of higher numbers of dead bacteria would be smaller sized aggregates formed by the remaining live bacteria. The CLSM images from the biofilms after the Live/Dead staining confirm this hypothesis (Figures 7a,b).

The number of the dead bacteria significantly increases when the concentration of Ag-BG in the specimens increases at 10 and 15 wt.%. The mechanism the antibacterial activity of Ag-BGCOMPs is based on the silver ions leaching, which takes place immediate after immersion and get enhanced with the time and the amount of Ag-BG. This mechanism can explain the fact that the formation of biofilm cannot be inhibited, however after a day of culture a higher number of bacteria are dead and this number becomes significant when the amount of Ag-BG in the composites is 10 wt.% or greater. It is expected that the number of dead bacteria could be further increased in the case of higher culture time as the amount of the released silver ions increases with the immersion time (Chatzistavrou et al., 2014). Furthermore, the antibacterial action of the specimens' extracts collected after 22 days, against two different bacteria strains confirms the long term antibacterial properties of the Ag-BGCOMP. The leaching antibacterial action is the main mechanisms of antibacterial activity, which seems to last at least 22 days. Taken together, we suggest that Ag-BGCOMP has far superior and long lasting anti-bacterial properties than the controls suggesting that Ag-BGCOMP can be instrumental in inhibiting secondary caries formation.

## Conclusions

The new bactericidal and bioactive composites Ag-BGCOMP were observed to be homogeneous, with significant bioactive properties. Enhanced remineralizing properties were observed through increased formation of apatite phase. The mechanical properties are not significantly affected after Ag-BG addition compared to control samples even after long period of time being immersed in medium. Finally, the inhibition of biofilm formation combined with long lasting antibacterial action could potentially suggest the suitability of Ag-BGCOMP for dental applications. This study significantly advances the research toward to the development of novel materials that can reduce risk factors and potentially halt the caries decay, while stimulating remineralization.

## Author contributions

XC: Substantial contributions to the conception, design of the work, analysis and interpretation of data for the work. Drafting the work. AL: Substantial contributions to the acquisition and analysis of data for the work. Drafting the work. LP: Substantial contributions to the acquisition and interpretation of data for the work. Revising the work critically for important intellectual content. EP and KP: Substantial contributions to the interpretation of data. Revising the work critically for important intellectual content. JF: Substantial contributions to the acquisition and interpretation of data. Revising the work critically for important intellectual content. SF: Substantial contributions to the conception, design, acquisition and interpretation of data for the work. Revising the work critically for important intellectual content. CG-C and NK: Substantial contributions to the conception, design and interpretation of data for the work. Revising the work critically for important intellectual content. PP: Substantial contributions to the conception, design, analysis and interpretation of data for the work. Revising the work critically for important intellectual content.

### Conflict of interest statement

The authors declare that the research was conducted in the absence of any commercial or financial relationships that could be construed as a potential conflict of interest. The reviewer AS and handling Editor declared their shared affiliation.
